# Editor's Message

**DOI:** 10.29173/jchla29717

**Published:** 2023-08-01

**Authors:** Colleen Pawliuk

**Affiliations:** Rédactrice en chef, JABSC / JCHLA

*[Français en suite]*

Welcome to the August 2023 Issues of JCHLA/JABSC. I hope everyone is enjoying the summer weather. I am excited to share that JCHLA/JABSC is now indexed in Web of Science Core Collection as part of the Emerging Sources Citation Index (ESCI). ESCI was added to Web of Science Core Collection in 2022. Not only does this aid in discoverability of the works published in JCHLA/JABSC, but beginning in 2024, JCHLA/JABSC will also be assigned a Journal Impact Factor.

I am also very proud to share the outcomes of our work this year with Equity, Diversity, and Inclusion. Firstly, the JCHLA/JABSC Editorial Team has developed a name change policy. This policy allows authors to request a name change for any reason. You can read the full policy on our website. Secondly, JCHLA/JABSC will no longer be using the title “Editor-in-Chief” due to the significance of the term “chief” to Indigenous culture and its use as a pejorative term towards indigenous men. Moving forward JCHLA/JABSC will use the title “Editor” in its place. If you have interest in learning more about eliminating the term “chief” from professional titles you can read an excellent discussion of the issue here.

This issue marks the end of my three-year term on the JCHLA/JABSC Editorial Board. The experience has been extremely rewarding and I am grateful to all the other JCHLA/JABSC and CHLA/ABSC members who I have been so lucky to work with over the years. Starting in September, Megan Kennedy (University of Alberta) will assume the position of Editor and Jessica McEwan (University of Ottawa) the position of Senior Editor. We are happy also to welcome Ashley Farrell (University Health Network) as Junior Editor and Tyler Ostapyk (University of Manitoba) as Production Editor.

The August 2023 issue includes one research article, “Scaffolded, embedded required: information literacy education in undergraduate health sciences.” Also included are three book reviews related to systematic reviews and a product review of ResearchRabbit, a literature discovery tool supported by artificial intelligence. As always, we thank our authors, readers and reviewers for their contributions and support!

**Colleen Pawliuk**

JCHLA/JABSC Editor-in-Chief

*Email: editor@chla-absc.ca*

Bienvenue au numéro d'août 2023 du JABSC / JCHLA. J'espère que tout le monde profite de l’été. Je suis ravie de partager que le JABSC / JCHLA est maintenant indexé dans Web of Science Core Collection via le Emerging Sources Citation Index (ESCI). L'ESCI a été ajouté à la collection de base de Web of Science en 2022. Non seulement cela aide à la découvrabilité des œuvres publiées dans le JABSC / JCHLA, mais à partir de 2024, le JABSC / JCHLA se verra également attribuer un facteur d'impact de journal.

Je suis également très fiere de partager les résultats de notre travail cette année avec l'équité, la diversité et l'inclusion. Premièrement, l'équipe éditoriale du JABSC / JCHLA a élaboré une politique de changement de nom. Cette politique permet aux auteurs de demander un changement de nom pour n'importe quelle raison. Vous pouvez lire la politique complète sur notre site Web. Deuxièmement, le JABSC / JCHLA n'utilisera plus le titre de « rédacteur en chef » en raison de l'importance du terme « chef » pour la culture autochtone et de son utilisation comme terme péjoratif envers les hommes autochtones. À l'avenir, le JABSC / JCHLA utilisera le titre « Éditeur » à sa place. Si vous souhaitez en savoir plus sur l'élimination du terme « chef » des titres professionnels, vous pouvez lire une excellente discussion sur la question ici.

Ce numéro marque la fin de mon mandat de trois ans au comité de rédaction du JABSC / JCHLA. L'expérience a été extrêmement enrichissante et je suis reconnaissante à tous les autres membres du JABSC / JCHLA et de l’ABSC / CHLA avec lesquels j'ai eu la chance de travailler au fil des ans. À compter de septembre, Megan Kennedy (Université de l'Alberta) occupera le poste de rédactrice et Jessica McEwan (Université d'Ottawa) le poste de rédactrice senior. Nous sommes également heureux d'accueillir Ashley Farrell (Réseau universitaire de santé) à titre de rédactrice junior et Tyler Ostapyk (Université du Manitoba) à titre de rédacteur de production.

Le numéro d'août 2023 comprend un article de recherche, « Scaffolded, embedded required: information literacy education in undergraduate health sciences ». Sont également inclus trois critiques de livres liées à des revues systématiques et une revue de produit de ResearchRabbit, un outil de découverte de littérature soutenu par l'intelligence artificielle. Comme toujours, nous remercions nos auteur.trice.s, lecteur.trice.s et réviseur.e.s pour leurs contributions et leur soutien!

**Colleen Pawliuk**

*Rédactrice en chef, JABSC / JCHLA*

*Courriel: editor@chla-absc.ca*

